# Editorial: Organ Modification for Edible Parts of Horticultural Crops

**DOI:** 10.3389/fpls.2019.00961

**Published:** 2019-07-23

**Authors:** Yuke He, Guusje Bonnema, Han Xiao, Yunde Zhao

**Affiliations:** ^1^National Key Laboratory of Plant Molecular Genetics, Shanghai Institute of Plant Physiology and Ecology, Chinese Academy of Sciences, Shanghai, China; ^2^Plant Breeding, Wageningen University and Research, Wageningen, Netherlands; ^3^Section of Cell and Developmental Biology, University of California, San Diego, San Diego, CA, United States

**Keywords:** morphological modification, corm, bulb, curd, fleshy fruit, fleshy root, leafy head, tuber

Plant development refers to various cellular processes that govern the morphogenesis of root, stem, leaf, flower, fruit, and seed. Plant development is regulated by genetic, epigenetic, and environmental factors. Developmental processes includes the determination, initiation, differentiation, and expression of different types of primordia on shoot/root apical meristems, and involve the establishment of polarity, cell division and differentiation of cell and tissue, phase transition, and hormone responses. Plant organs such as leaf, stem, root, flower, and fruit plays roles in water and mineral absorption, transportation, photosynthesis, pollination, fertilization, and other physiological processes. However, many horticultural crops often display enlarged organs to develop into the edible products: leafy head, bulb, tuber, fleshy stem, corm, fleshy root, root tuber, curds, and fleshy fruits. Unlike the grains of many field crops that mainly provide starch, the modified organs in horticultural crops store vitamins, secondary metabolites, minerals, and dietary fiber that are important for human health. Their commercial quality mainly depends on the diversity in size, shape, surface features, and texture. It is very interesting to know how these plant organs were selected and domesticated to become the edible parts of horticultural crops. Recently, molecular biology technology have been applied to address the key scientific questions and research directions with regard to morphological modifications of leaf, stem, root, flower, and fruit. For example, several miRNAs have been identified to play essential roles in determination of size, shape, and timing of leafy heads (Mao et al., 2014; Wang et al., 2014; Ren et al., 2018).

The chapters address genetic and molecular basis of morphological modifications of plant organs in horticultural crops. The focused topic issues cover dynamic phenotyping of the modified organs: leafy head, bulb, tuber, fleshy stem, corm, fleshy root, root tuber, curd, and fleshy fruit during plant development, genetic variation of modified organs, functional analysis of genes of organ-related traits that control size, shape, weight, texture, color and flavor of the modified organs, regulation of non-coding RNAs controlling morphological modification, effects of abiotic stress molecular mechanisms underlying metamorphosis of plant organs.

Genetic basis of leafy heads and bulbs is a critical topic. Many vegetable crops develop into certain types of leaf curvature required for high yield and quality. Recent research has revealed that miRNAs in *Brassica* crops regulate the timing and shape of leafy heads (Mao et al., 2014; Wang et al., 2014). Several papers in this issues described the origin, diversity, and development of leafy heads. Three papers (“Genetic analysis of Chinese cabbage reveals correlation between rosette leaf and leafy head variation” by Sun et al.; “Characterization of non-heading mutation in heading Chinese cabbage (*Brassica rapa* L. ssp. *pekinensis*)” by Li et al.; and “Transcription coactivator ANGUSTIFOLIA3 (AN3) regulates leafy head formation in Chinese Cabbage” by Yu et al.). These papers described the phenotyping, DNA resequencing and gene function identification of Chinese cabbage. Modification of flower organ is important for function transformation of edible organs. Two papers are related to flower organ modifications (“Differential gene expression caused by the F and M loci provides insight into ethylene-mediated female flower differentiation in cucumber” by Pan et al. and “defective apetala2 genes lead to sepal modification in Brassica crops” by Zhang et al.). Shape, size, color, and flavor of fleshy fruits are genetically related to leaf features. One paper “Chemical composition and crystal morphology of epicuticular wax in mature fruits of 35 pear (*Pyrus* spp.) cultivars” by Wu et al. “discloses crystal morphology of “epicuticular wax in mature fruits;”” and another paper “Functional dissection of Auxin Response Factors in regulating tomato leaf shape development” by Wu et al. provide the evidences that auxin response factors regulate tomato leaf shape and fruit development. The paper “Plant organ shapes are regulated by protein interactions and associations with microtubules” addressed the importance of microtubules to formation of organ shape (Lazzaro et al.). Finally, this book contains eight papers from scientists working in institutions from different countries. Thus, we can claim that this book, with its multiple scientific voices, provides also a contribution to understanding genetic basis and molecular mechanisms underlying organ modification.

## Author Contributions

All authors listed have made a substantial, direct and intellectual contribution to the work, and approved it for publication.

### Conflict of Interest Statement

The authors declare that the research was conducted in the absence of any commercial or financial relationships that could be construed as a potential conflict of interest.
